# The role of JAK/STAT/SOCS3 signaling in rats with brain damage induced by early alcohol exposure after birth

**DOI:** 10.1002/pdi3.64

**Published:** 2024-01-10

**Authors:** Chen Yang, Jianxiong Gui, Dishue Huang, Ran Ding, Jie Liu, Wenjie Zhao, Jing Yang, Ziyao Han, Lingling Xie, Xiaoyue Yang, Yanan Pan, Mingdan Xie, Li Cheng, Xiaojie Song, Li Jiang

**Affiliations:** ^1^ Department of Neurology Children's Hospital of Chongqing Medical University National Clinical Research Center for Child Health and Disorders Ministry of Education Key Laboratory of Child Development and Disorders Chongqing Key Laboratory of Pediatrics Chongqing China; ^2^ Chongqing Mental Health Center Chongqing China; ^3^ Chengdu Women and Children Central Hospital Chengdu Sichuan China; ^4^ Zhejiang University School of Medicine Children's Hospital Hanzhou Zhejiang China

**Keywords:** alcohol exposure, brain damage, JAK/STAT/SOCS3, neonatal rats, neuroinflammation

## Abstract

Early postnatal alcohol exposure can have negative impacts on neonatal rat brain development and function. Our research explored the impacts of alcohol exposure from postnatal day 4 to PD9 on Sprague‒Dawley rat pups. Pups were intragastrically administered with either an alcohol milk solution or a pure milk solution twice daily. On PD10, brains were analyzed via histological and biochemical methods. Alcohol exposure led to growth impairment, behavioral abnormalities, and cognitive deficits. It also reduced microglial numbers in the hippocampus while activating the remaining microglia to secrete IL‐6. In addition, alcohol induced the upregulation of pro‐apoptotic factors and downregulation of the anti‐apoptotic protein BCL‐2 in the hippocampus by activating the JAK/STAT/SOCS3 signaling pathway. Similar effects were observed in vitro when BV‐2 cells were exposed to ethanol and HT‐22 cells were exposed to IL‐6. The drug AG490, a STAT3 inhibitor, mitigated IL‐6‐induced JAK/STAT activation and neuronal apoptosis in HT‐22 cells. Overall, these findings demonstrate that early‐life alcohol exposure triggers an inflammatory microglial response involving the release of IL‐6, which activates JAK/STAT signaling, leading to hippocampal neuronal apoptosis and developmental/cognitive impairments. AG490 may disrupt this inflammatory signaling cascade and cause neuronal damage.

## Graphical abstract

Early postnatal alcohol exposure can promote microglial activation, increase IL‐6 secretion, activate the JAK/STAT signaling pathway, and subsequently lead to hippocampal cell apoptosis both in vitro and in vivo. AG490 may inhibit the activation of the JAK/STAT/SOCS3 signals, thereby alleviating microglial cell activation, reducing IL‐6 secretion, and decreasing neuronal apoptosis.

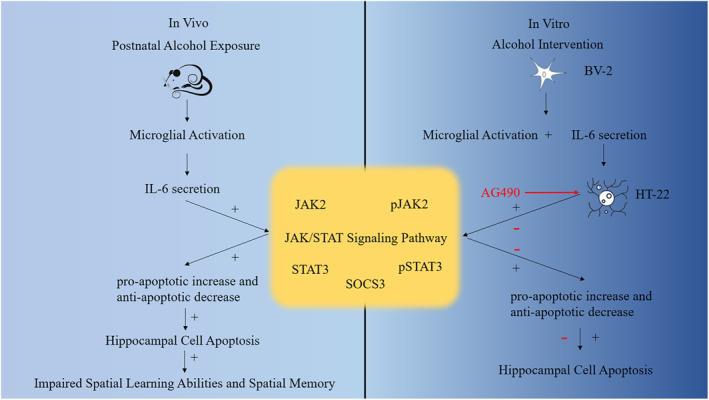

## 1 INTRODUCTION

Fetal alcohol spectrum disorder (FASD) is a diagnostic term used to describe fetal alcohol‐induced effects that include physical abnormalities as well as cognitive and/or behavioral impairments.^1^, ^2^, ^3^, ^4^ A wide spectrum of symptoms corresponding to FASD, such as mild craniofacial abnormalities, growth retardation, neurological disorders, behavioral and cognitive impairment, and birth deformities.^5^ Epidemiological studies have shown that FASD is a worldwide problem, and the overall prevalence of FASD is 1.06‐113.22/1000 people.^1^, ^5^

However, the pathogenesis of FASD is still unclear, and there is currently no effective treatment available. Previous research has indicated that alcohol, a teratogenic drug, significantly affects the connection between neurons and the generation of neurotransmitters. This leads to the death of neurons and the development of FASD.^6^, ^7^, ^8^ Nevertheless, thorough research on the neuroimmune system has revealed that neuroinflammation is the typical pathogenic trait brought on by alcohol misuse.^9^, ^10^, ^11^, ^12^

The neuroimmune system relies primarily on microglial activation and cytokine secretion to exert its effects. According to related research, proinflammatory cytokines like interleukin‐6 (IL‐6), IL‐1, and tumor necrosis factor‐α (TNF‐α) are secreted by activated microglia.^13^, ^14^, ^15^ These factors can lead to neural cell death and impair brain function and development. Under normal conditions, IL‐6 expression in brain tissue is very low and almost undetectable. However, IL‐6 levels are markedly increased following brain injury.^16^ Nowadays, the majority believe that the central nervous system (CNS) primary source of IL‐6 is microglia.^15^, ^17^ Research has demonstrated that in individuals suffering from severe traumatic brain injury, IL‐6 is a significant indicator of pathogenic processes during the acute phase.^17^ Furthermore, an increasing mount of research indicates that IL‐6 is significantly related to neurological diseases and associated with alteration to cognitive function and behavior.^18^, ^19^ The massive secretion of IL‐6, an inflammatory factor, can lead to extensive activation of JAK2/STAT3.^20^, ^21^ The JAK/STAT signaling pathway mainly mediates cell proliferation, differentiation, migration, and apoptosis.^22^, ^23^ Studies on other neurological disease models have shown that when the nervous system is experiencing ischemia and hypoxia, triggering the JAK/STAT pathway stimulates microglial activation, resulting in neuronal death.^24^ Moreover, suppressor of cytokine signaling 3 (SOCS3), an antagonistic JAK/STAT pathway regulator, is secreted in reaction to activated JAK2/STAT3. This leads to an increase in programmed cell death and a decrease in the formation of new brain synapses, aiming to preserve homeostatic balance.^25^ Downregulation of the JAK/STAT pathway can inhibit abnormal microglial activation and ameliorate neuroinflammatory injury.^26^ Current research has shown that AG490 has a chemical structure similar to that of tyrosine. It competes with tyrosine kinases for binding sites, inhibiting JAK2/STAT3 phosphorylation in microglia, thereby reducing cell apoptosis and inflammatory responses. It can also mitigate blood‒brain barrier disruption mediated by endothelial cells.^27^, ^28^

To investigate potential structural brain damage induced by FASD, we explored the effects of early postnatal alcohol exposure on rat brain structure in this study. In vitro experiments will be conducted using alcohol‐treated microglia to simulate the impacts of activated microglia on neurons. By employing this strategy, we can examine the function of the JAK/STAT signaling pathway in the context of early postnatal alcohol exposure, enhancing our understanding of the associated pathogenic mechanisms following such exposure in early life.

## 2 MATERIALS AND METHODS

### 2.1 Animals and treatments

The Experimental Animal Center of Chongqing Medical University (Chongqing, China) provided the Sprague‐Dawley (SD) rats (230–250 g) (animal experimentation premission number: CQLA‐2014‐0651). The animal procedures were conducted in compliance with the guidelines established by the Experimental Animal Management and Use Committee of Chongqing Medical University. The rats used selected for this study were housed in a controlled environment that was free from pathogens. They were exposed to a 12‐h light/12‐h dark cycle and maintained at a humidity level of 55 ± 5% at 25 ± 1°C. Additionally, they had unrestricted utilization of water and food. They spent seven days getting used to the dwelling chamber before breeding. Once vaginal plugs were found, each pregnant rat was placed inside a single cage to give birth naturally.

Every litter was culled at postnatal day (PD) 3, when there were nine pups (5 males, and 4 females, if possible). In postnatal day 4 (PD4), we employed random assignment to allocated the pups from a solitary litter into two distinct experimental groups: alcohol‐exposed (AE) groups and normal control (NC). To identify the pups, we applied colored nontoxic black ink on their paws. Expect the intragastric intubation, the pups and the dams were left alone until PD10, whereupon they were sacrificed. For the data shown here, a total of 82 pups from 11 litters were used. Specifically, for body and brain weight comparisons, 20 AE pups and 20 NC pups were used. For Enzyme‐linked immunosorbent assay (ELISA), 5 AE pups and 5 NC pups were used. For Western blotting, 10 AE and 10 NC pups were used. For immunofluorescence, 5 AE and 5 NC pups were used. For HE, 5 AE and 5 NC pups were used. For the Morris water maze (MWM), 16 AE, and 16 NC pups were used.

### 2.2 Cell culture

BV2 cells were purchased from Procell Life Science & Technology Co., Ltd. (China). BV2 cells were placed in a culture dish following the experimental procedures and grown in DMEM medium containing 10% fetal bovine serum and 1% GlutaMAX. The cells were incubated at a temperature of 37°C with a CO_2_ concentration of 5%. After cell adherence, ethanol was administered at final concentrations of 0, 50, 100, 200, or 400 mM for 1 or 2 h of treatment. The cell viability in each group was measured by adding 10 μl of CCK8 solution into each well, gently shaking the plate, and incubating the mixture for 3–4 h at 37°C with 5% CO_2_ until the medium changed color to orange. The optical density (OD) for each well was determined at 450 nm via a microplate reader, with 630 nm serving as the reference wavelength. The cell viability (%) of each group was calculated as follows: relative absorbance × 100% = OD value of each group/OD value of control group × 100%. HT‐22 cells were purchased from Procell Life Science & Technology Co., Ltd. (China). HT‐22 cells were placed in a culture dish following the experimental procedures and grown in DMEM with 10% fetal bovine serum and 1% GlutaMAX. The cells were incubated at a temperature of 37°C with a CO_2_ concentration of 5%. After cell adherence, IL‐6 intervention and IL‐6 inhibitor experiments were performed at predetermined concentrations of 0, 20, 40, 60, and 40 ng/ml + AG490 for 24 h of treatment.

### 2.3 Alcohol exposure paradigm

The AE pups received intragastric intubation on PD4‐9 and were given 5.25 g/kg/day of alcohol in a milk formula (11.9% v/v) in 2 doses spaced 2 h apart.^29^, ^30^ Two and four hours after the second alcohol does on PD4, the AE pups additionally got two milk‐only feeds as a calorie supplement; on PD 5–9, the AE pups received one milk‐only feed 2 h after the second alcohol dose. The NC pups received pure milk. However, both the AE and NC pups were separated from the dam only for the purpose of intragastric administration and weighing to ensure proper development.

### 2.4 HE staining

On PD10, 5 rats from each group were assigned at random for hematoxylin‐eosin (HE) staining. The rats' hippocampal organotypic tissues were collected, fixed in 4% paraformaldehyde for 24 h and generally processed into 5‐μm thick paraffin‐embedded slices. All slices were put on slides that had been pretreated with 3‐aminopropyl‐triethoxysilane and dried for 15 min in a 37°C oven. HE staining was performed using conventional methods. Deparaffinized sections were treated with a series of procedures for HE staining, which included 45 s of hematoxylin solution incubation, 3 s of 0.5% HCl differentiation, 10 s of lithium carbonate solution, and 45 s of eosin staining solution counterstaining. Sections were observed under a light microscope (Eclipse 55i; Nikon Corporation, Tokyo, Japan).

### 2.5 Enzyme‐linked immunosorbent assay

ELISA was used to measure the concentrations of IL‐6 in the hippocampus and cell culture media. The rats were sacrificed, and hippocampal tissue was rapidly dissected. The hippocampus tissues were crushed using a glass homogenizer while cold on ice. A homogenization buffer consisting of 0.01 M phosphate‐buffered saline (PBS) with a  pH of 7.4 was employed. The resulting mixture was then centrifuged at 4°C for 15 min at 12,000 rpm. The supernatants were collected and preserved at −80°C until use. The IL‐6 ELISA kits from Dakewe Biotech were utilized in accordance with the maunfacturer's instructions. The samples were analyzed in duplicate, and the results are shown as pg/mg protein. The cell samples were treated in the same way.

### 2.6 Western blot

On PD 10, the rats' hippocampi were swiftly extracted and transferred to liquid nitrogen preservation. The cell proteins were also collected for Western blot analysis after culture. The Pierce bicinchoninic acid protein assay kit (Thermo Scientific) was used to measure the protein concentration. The extracts included 40 μg of total protein and were separated on 10%–15% sodium dodecyl sulfate (SDS) polyacrylamide gels. Following separation, the protein were transferred to polyvinylidene difluoride membranes with a pore size of 0.22 μm (Millipore Corp.). After a 90 minuted blocking period in TBST with 5% fatty‐free BSA (meilunbio), the membranes were subjected to incubation overnight at 4°C with primary antibodies mentioned below: anti‐SOCS‐3 (rabbit monoclonal antibody; Abcam, 1:1000), anti‐BCL‐2/BAX/CASPASE‐3, anti‐JAK2/pJAK2/STAT3/pSTAT3 and anti‐β‐actin antibodies (1:500, rat monoclonal antibody; Boster, 1:500). The following day, the membranes were incubated for 90 min at room temperature with the matching secondary antibody (anti‐rabbit/rat IgG; ZSGB‐BIO, 1:5000). The protein bands were observed with a Bio‐Rad Imager following the use of a clear western electrochemiluminescence substrate (Bio‐Rad). Density measurement was used to assess the protein band intensity using Syngene imaging systems.

### 2.7 Immunofluorescence

The rat brains were sliced into equential frozen coronal slices with a thickness of 30 μm. These slices were then preserved in a cryoprotectant solution at a temperature of 4°C until the immunofluorescence staining procedure was carried out. The following primary antibodies were used: anti‐CASPASE‐3 (rabbit monoclonal antibody, Abcam, 1:50), anti‐NEUN (mouse monoclonal antibody, CST, 1:100), anti‐NF200 (mouse monoclonal antibody, Proteintech, 1:50), anti‐Iba‐1 (rabbit monoclonal antibody, CST, 1:100), and DAPI. All the antibodies used were incubated with the tissue samples overnight. A confocal microscope with laser scanning was used to capture all of the pictures. Quantification of the number of NeuN‐positive cells in the hippocampal CA1, CA3, and dentate gyrus (DG) regions was performed. The fluorescence intensity of CASPASE‐3 positive regions was measured using ImageJ software (NIH Image; US National Institutes of Health, Bethesda, MD, USA).

### 2.8 Morris water maze

To investigate learning and memory capacity, 16 selected random rats from each group were put through the Morris water maze (MWM) test at PD28. A 75 cm radius round fiberglass water tank that had been intentionally separated into four sections (N, E, S, and W) served as the MWM apparatus. The water tank's black platform (7 cm radius) was dimmersed in opaque and warm (25 ± 1°C) water. The animals were given 60 s in the water tank on the first day to get used to the maze. After that, the acquisition test was run from the second to the sixth day. Every rat went through training in four trials per day over five days in a row. For each trial, the rats were placed in one of the four sections of the water tank and allowed to swim until they found the hidden, submerged goal platform, where they stayed for 3 s. A stick was used to guide the rats to the platform, and if they failed to locate it in 1 min, they were held there for 15 s. The ANY‐Maze video tracking system captured the escape latency. The platform was taken away on day 7, then the rats were placed into the water tank for a 60 s probe trail. The average number of platform crossings and the average duration of time spent in the target quadrant, where the platform was previously located were recorded by the ANY‐Maze video monitoring system.

### 2.9 Statistical analysis

The statistical analysis was performed using SPSS 17.0 statistical software. One‐way analysis of variance (ANOVA) was used to assess the body weight at PD4 and PD10, the ELSIA, and the densitometric western blot data with Bonferroni post hoc correction coming after. For image analysis, a minimum of four successive coronal sections from each rat were examined. The means ± SDs represent the results, which were analyzed statistically using one‐way ANOVA and post hoc multiple pairwise comparisons using the log‐rank test or the Bonferroni correction. All the data are presented as the means ± SEMs. Differences were considered significant if *p* < 0.05.

## 3 RESULTS

### 3.1 Weight measurements

**FIGURE 1:**
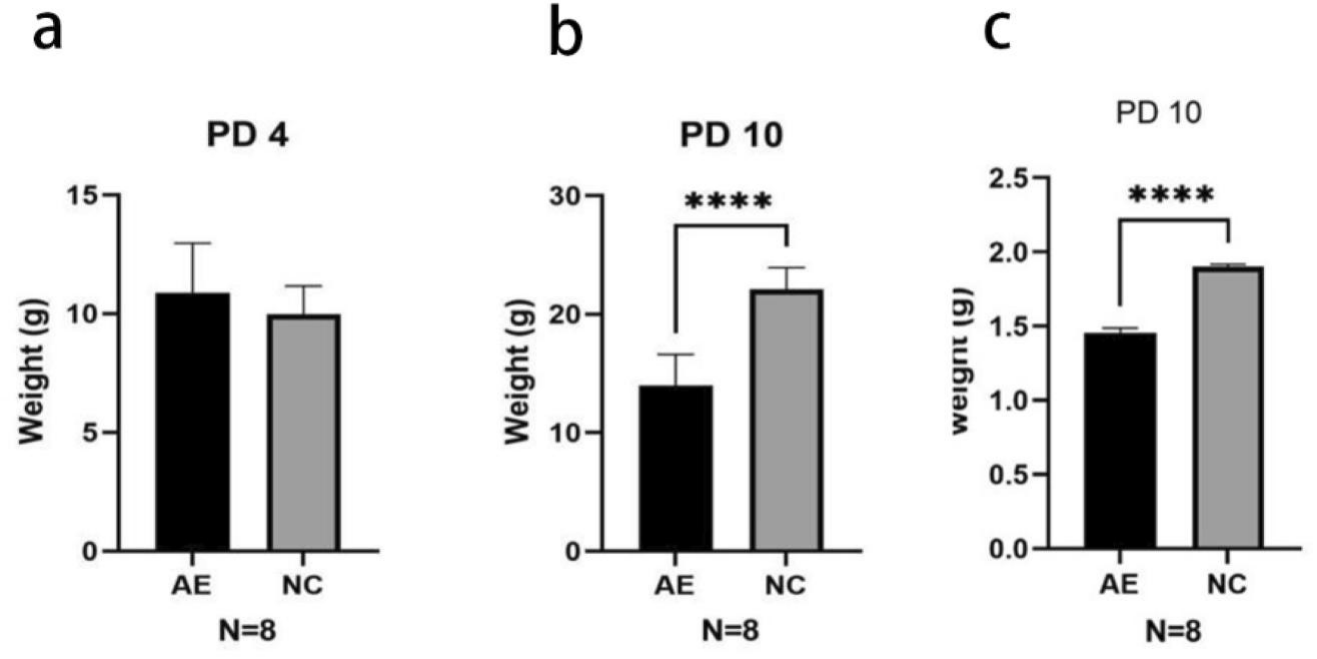
(A, B) Body weight data at PD4 and PD10 in the AE group and NC group. (C) Brain weight data at PD10 in the AE group and NC group. ****p < 0.0001.

Twenty rats in each group were weighed on PD4, and 20 rats were weighed on PD10. The body weight of the AE group on PD4 was 10.88 ± 2.09 g, and the weight of the NC group was 11.15 ± 1.95 g. The body weight of the AE group on PD10 was 3.96 ± 2.64 g, and the weight of the NC group was 22.10 ± 1.84 g. In comparison to the NC group, the AE group's body weight on PD10 was significantly lower (*p* < 0.0001). The brain weight of the AE group on PD10 was 1.457 ± 0.029 g, and the brain weight of the NC group was 1.902 ± 0.014 g. On PD10, the AE group's brain weight was significantly lower than the NC group's (*p* < 0.0001) (Figure 1).

### 3.2 HE staining

**FIGURE 2:**
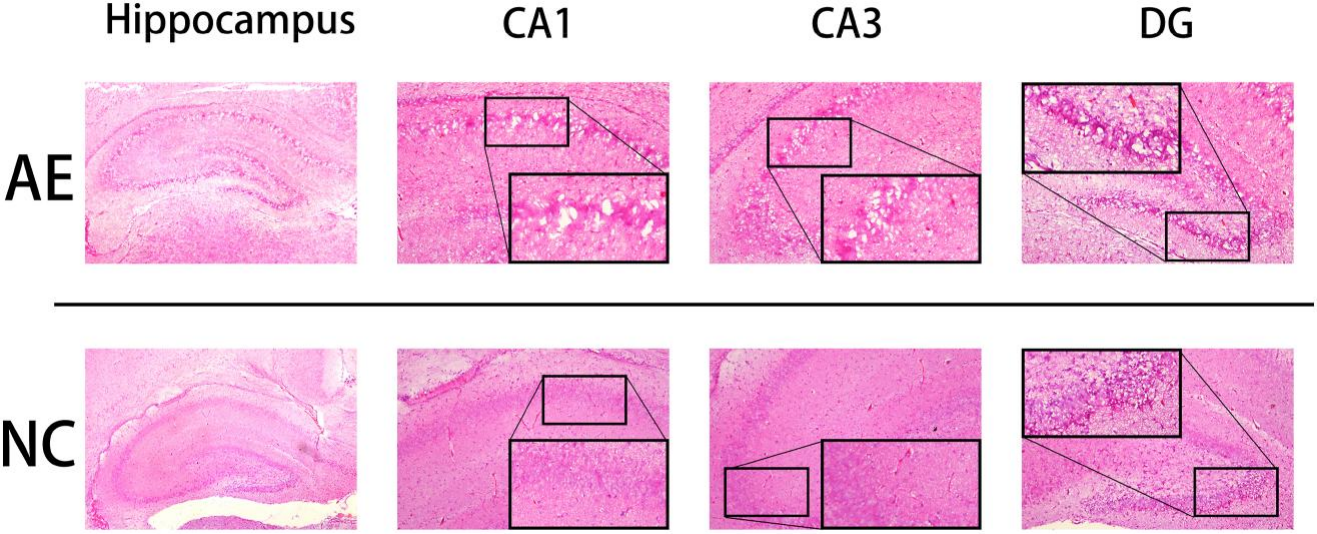
H&E staining of the hippocampus (40×). The structures of the CA1, CA3, and DG regions are shown on the right (100×). In these images, the areas enclosed by the black box were amplified to clearly delineate the structure.

Sprague–Dawley rats were exposed to alcohol from PD 4 to PD9. Hematoxylin and eosin (H&E) staining was used to observe hippocampal changes on PD10 in the two groups. As shown in Figure 2, in the hippocampus of NC rats, the neurons were arranged densely and regularly, with uniform cytoplasmic staining and clearly defined nuclear morphology. Compare them to the NC group members, the hippocampi of AE rats exhibited partial loss of neurons, sparse and irregular arrangement, disorganized neurons, cytoplasmic shrinkage and deep red staining, partial cell vacuolization, and pyknotic and hyperchromatic nuclei.

### 3.3 CCK8

**FIGURE 3:**
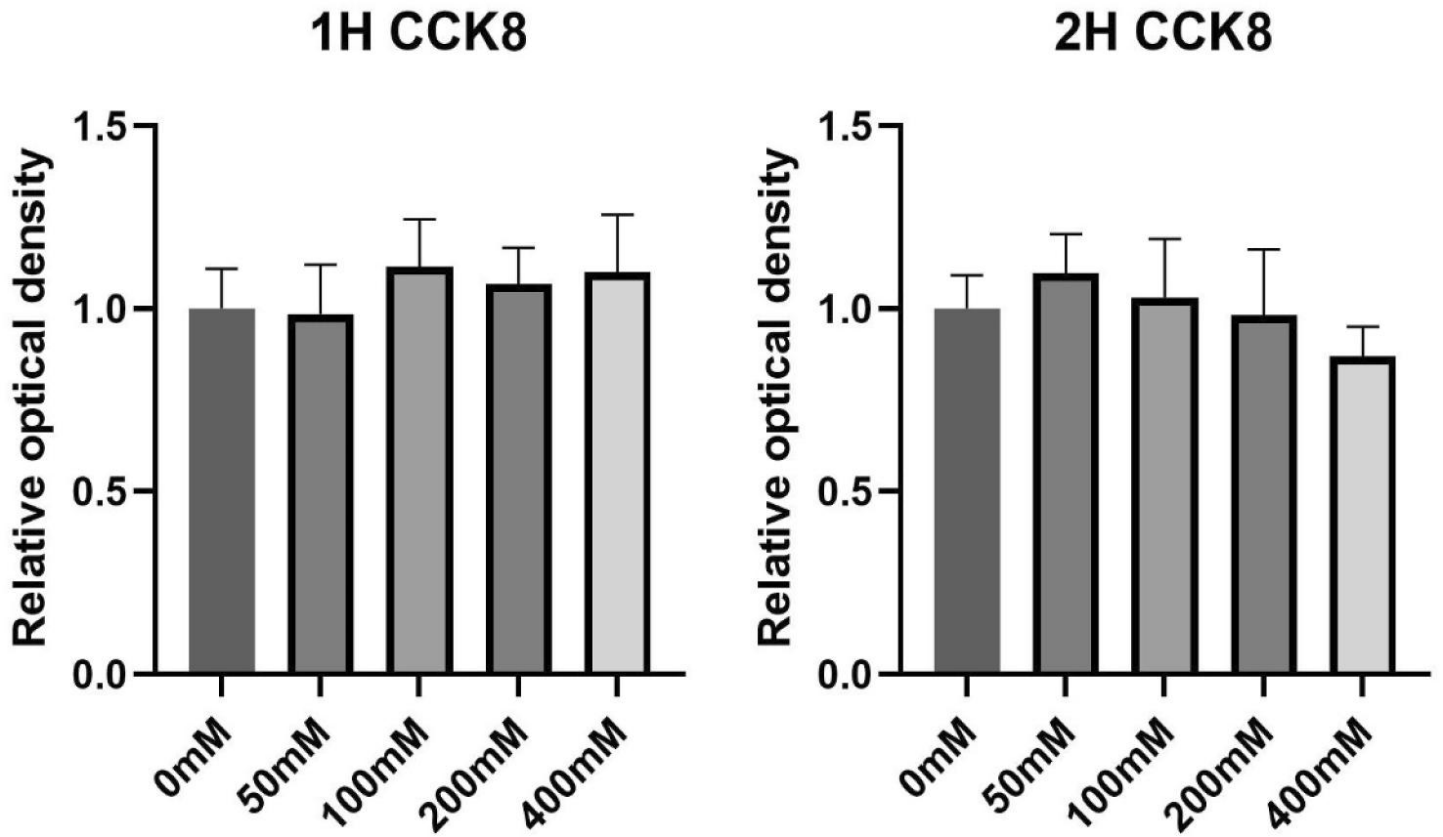
Microglial activity after treatment with different alcohol concentrations for different durations. The left bar graphs show the BV‐2 cell activity after treatment with alcohol at different concentrations for 1 h. The right bar graphs show the BV‐2 cell activity after treatment with different concentrations of 2H alcohol.

We conducted an in vitro analysis of the viability of BV‐2 cells exposed to different alcohol concentrations (0, 50, 100, 200, and 400 mM). To quantify the changes in viability, we added CCK8 reagent and used a multifunctional microplate reader to measure the optical density (OD) values of each well. The viability of microglia in the intervention groups treated with varying alcohol concentrations did not significantly change after 1 h of alcohol intervention. After 2 h of alcohol treatment, microglial viability decreased with increasing alcohol concentration, but these differences were not statistically significant (*p* > 0.05) (Figure 3).

### 3.4 Enzyme‐linked immunosorbent assay

**FIGURE 4:**
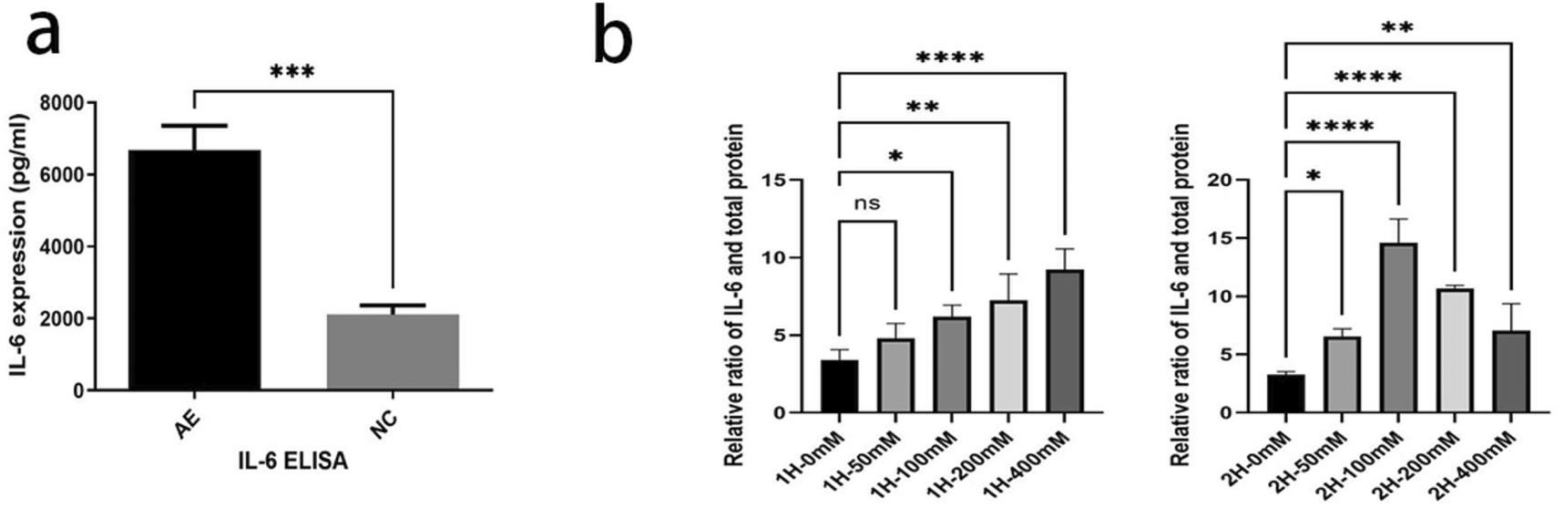
(A) Comparison of IL‐6 expression levels in the hippocampi of newborn rats on PD10 between the AE group and NC group, *n* = 5. (B) The expression level of IL‐6 in BV‐2 cells after treatment with various alcohol concentrations for 1 or 2 h. (C) Table 1. Expression level of IL‐6 in BV‐2 cells after treatment with different alcohol concentrations for 1 or 2 h ($\bar{x}$±SD); *n* = 3. *p < 0.05, **p < 0.01, ***p < 0.001, and ****p < 0.0001.

After early postnatal alcohol exposure in rats, ELISA was utilized to measure changes in IL‐6 expression in the hippocampus on PD10. The results showed that IL‐6 levels were significantly greater in the AE group (6686 ± 1493 pg/mL) than in the NC group (2112 ± 562.3 pg/mL) (*p* < 0.001) (Figure 4A). ELISA was also used to detect changes in IL‐6 expression in the culture medium of BV2 cells (Figure 4B,C). Following a one‐hour ethanol treatment, BV‐2 cells secreted more IL‐6 as the ethanol concentration rose; these differences were significantly elevated when compared to the control group's ethanol treatment at 100, 200, or 400 mM (*p* < 0.05). After 2 h of ethanol treatment, there was a significant rise in IL‐6 secretion at all doses when compared to the control group (*p* < 0.05). However, the IL‐6 levels did not further increase with increasing ethanol concentration. IL‐6 secretion peaked in the 100 mM ethanol treatment group and then gradually decreased as the ethanol concentration increased.

**TABLE 1:** Expression level of IL‐6 in BV‐2 cells after intervention with different alcohol concentrations for 1 or 2 h ($\bar{x}$±SD).

	0 mM	50 mM	100 mM	200 mM	400 mM
1 h	3.406 ± 0.660	4.790 ± 0.965	6.188 ± 0.739*	7.240 ± 1.699**	9.211 ± 1.347****
2 h	3.272 ± 0.256	6.564 ± 0.653*	14.610 ± 2.035****	10.680 ± 0.257****	7.064 ± 2.293**

### 3.5 Western blot

#### 3.5.1 Protein levels in the hippocampus of rats

**FIGURE 5:**
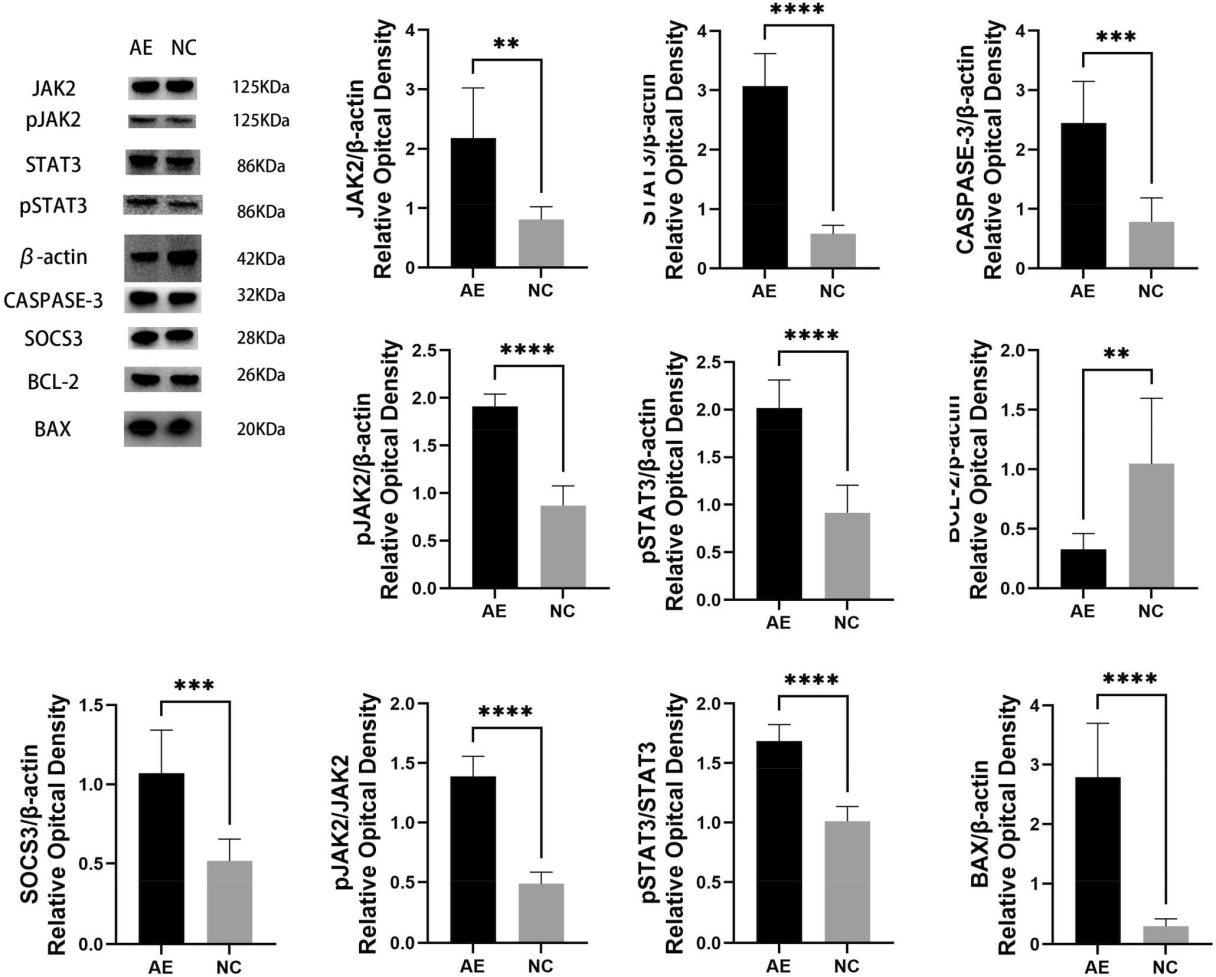
Protein expression levels of BAX, BCL‐2, SOCS3, CASPASE‐3, JAK2, pJAK2, STAT3, and pSTAT3 in the hippocampus of rats; **p < 0.01, ***p < 0.001, and ****p < 0.0001.

Western blotting was used to detect the protein expression levels of BAX, BCL‐2, and CASPASE‐3 in the hippocampus of the rats. As shown in Figure 5, immunopositive bands for BAX, BCL‐2, and CASPASE‐3 were single bands at approximately 20, 26, and 32 kDa, respectively. The ratios of the BAX, BCL‐2, and CASPASE‐3 band intensities to the corresponding internal reference band β‐actin were calculated. (a) Compared to those in the NC group, the protein expression of BAX and CASPASE‐3 was significantly greater in the AE group (*p* < 0.0001, *p* < 0.001). (b) BCL‐2 protein expression was markedly lower in the AE group than in the NC group (*p* < 0.01).

Furthermore, to ascertain whether the JAK/STAT signaling pathway was activated in the rats' hippocampal regions, Western blotting was employed to identify variations in the expression of proteins connected to the JAK/STAT‐SOCS3 signaling pathway. As shown in Figure 5, immunopositive bands for JAK2, pJAK2, STAT3, pSTAT3, and SOCS3 were single bands at approximately 125, 125, 86, 86, and 28 kDa, respectively. The ratios of the JAK2, pJAK2, STAT3, pSTAT3, and SOCS3 band intensities to the corresponding internal reference band β‐actin, as well as the pJAK2/JAK2 and pSTAT3/STAT3 ratios, were calculated. (a) Compared to those in the NC group (NC group), the expression of JAK2, pJAK2, STAT3, pSTAT3, and SOCS3 was markedly increased in the alcohol exposure group (AE group), and the differences across the two groups were statistically significant (*p* < 0.01). (b) The pJAK2/JAK2 and pSTAT3/STAT3 ratios were significantly greater in the AE group than in the NC group (*p* < 0.0001) (Figure 5).

#### 3.5.2 Protein expression levels in the cell lines

**FIGURE 6:**
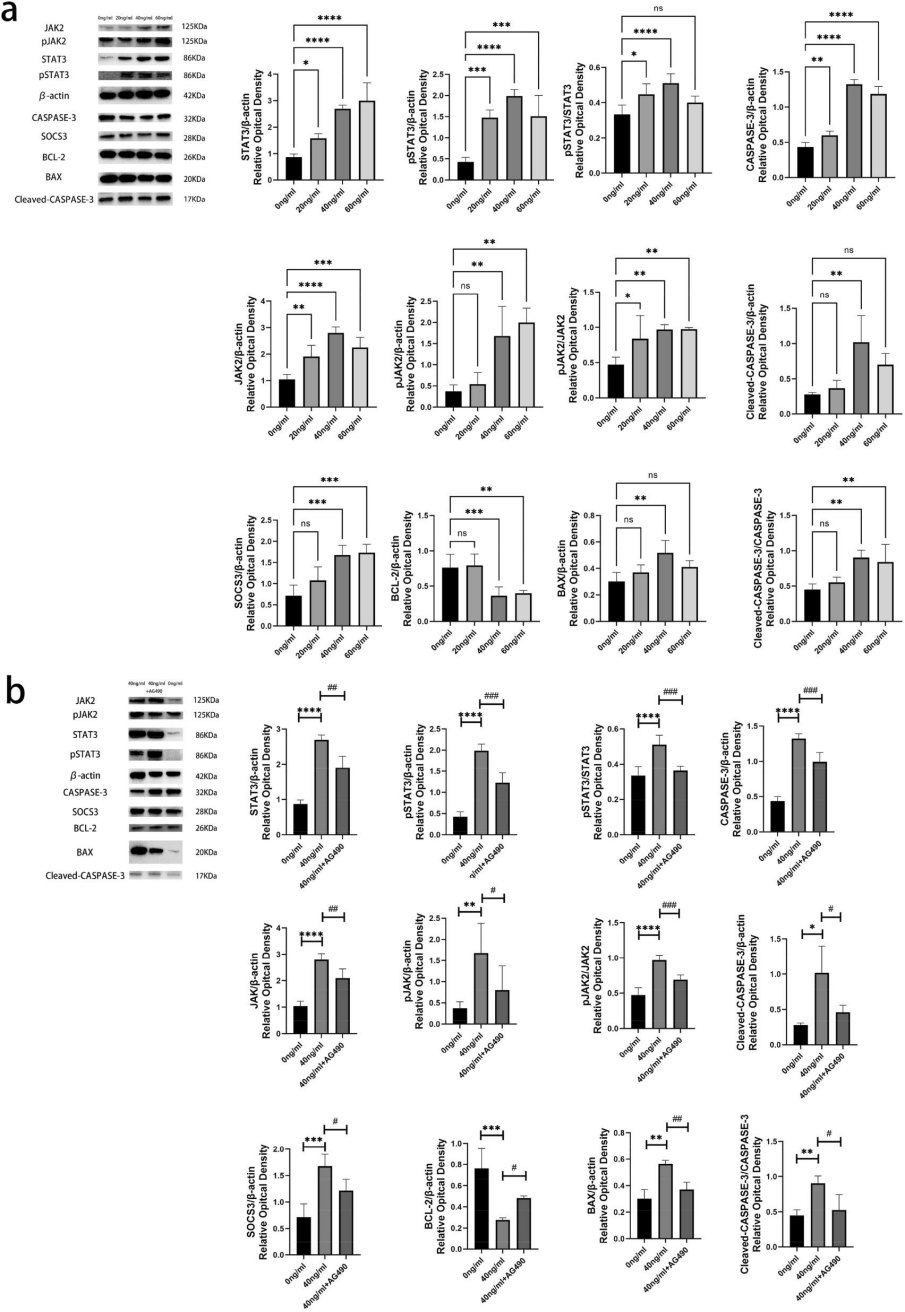
(A) The protein expression levels of JAK2, pJAK2, STAT3, pSTAT3, SOCS3, BAX, BCL‐2, and CASPASE‐3 in HT‐22 cell culture samples; *n* = 3. (B) Protein expression levels of JAK2, pJAK2, STAT3, pSTAT3, SOCS3, BAX, BCL‐2, and CASPASE‐3 in HT‐22 cell culture samples; *n* = 3; compared with the 0 ng/mL group; *p < 0.05, **p < 0.01, ***p < 0.001, and ****p < 0.0001; compared with the 40 ng/mL group; ^#^ *p* < 0.05, ^##^ *p* < 0.01, and ^###^ *p* < 0.001.

Western blot analysis was also used to detect the expression of JAK2, pJAK2, STAT3, pSTAT3, and SOCS3 in the cell culture samples (including cells and cell culture media) (Figure 6A). The ratios of the JAK2, pJAK2, STAT3, pSTAT3, and SOCS3 band intensities to the corresponding β‐actin, as well as the pJAK2/JAK2 and pSTAT3/STAT3 band intensities, were calculated. (a) Compared to those in the untreated control (0 ng/mL IL‐6), the protein levels of JAK2, pJAK2, STAT3, pSTAT3, and SOCS3 in HT‐22 cells increased to varying extents after treatment with different concentrations of IL‐6. STAT, pSTAT, and JAK protein levels increased significantly after treatment with 20, 40, or 60 ng/mL IL‐6 (*p* < 0.05). pJAK and SOCS3 protein levels increased significantly after 40 or 60 ng/mL IL‐6 treatment (*p* < 0.001). (b) After 40 ng/mL IL‐6 treatment, the protein levels of JAK2, pJAK2, STAT3, pSTAT3, and SOCS3 in the cell culture samples were significantly greater than those in the untreated control (0 ng/mL) samples (*p* < 0.01). (c) The pJAK2/JAK2 and pSTAT3/STAT3 ratios were significantly greater in the IL‐6 treatment groups than in the untreated control group (0 ng/mL) (*p* < 0.05). d) For the HT‐22 cells treated with 40 ng/mL IL‐6, the protein levels of JAK2, pJAK2, STAT3, pSTAT3, and SOCS3 in the cell culture samples decreased significantly to varying extents after the addition of the STAT3 inhibitor AG490 (*p* < 0.05). (e) The pJAK2/JAK2 and pSTAT3/STAT3 ratios were significantly lower in HT‐22 cells after treatment with 40 ng/mL IL‐6 and AG490 (*p* < 0.001) (Figure 6B).

Western blotting was used to detect the expression levels of BAX, BCL‐2 and CASPASE‐3 in the cell samples (Figure 6A). The statistical results are: (a) The CASPASE‐3 protein levels in the groups treated with different concentrations of IL‐6 were significantly higher than those in the untreated control group (0 ng/ml IL‐6) (*p* < 0.01). (b) The protein levels of cleaved CASPASE‐3 and BAX were significantly higher in the treated group (40 ng/ml or 60 ng/ml) than in the untreated control group (*p* < 0.01). (c) The BCL‐2 protein levels following 40 ng/ml or 60 ng/mL IL‐6 treatment were significantly lower than those in the untreated control group (0 ng/ml IL‐6) (*p* < 0.001). (d) After 40 or 60 ng/mL IL‐6 treatment, the Cleaved‐CASPASE‐3/CASPASE‐3 ratio markedly increased (*p* < 0.01). e). As the aforementioned parameters demonstrated the most significant changes under treatment with 40 ng/mL IL‐6, this concentration of IL‐6 was selected for combination treatment with AG490. Among the groups treated with 40 ng/mL IL‐6, the addition of the STAT3 inhibitor AG490 significantly decreased the expression of BAX and CASPASE‐3 (*p* < 0.01) and markedly increased BCL‐2 expression (*p* < 0.05) (Figure 6B).

### 3.6 Immunofluorescence

**FIGURE 7:**
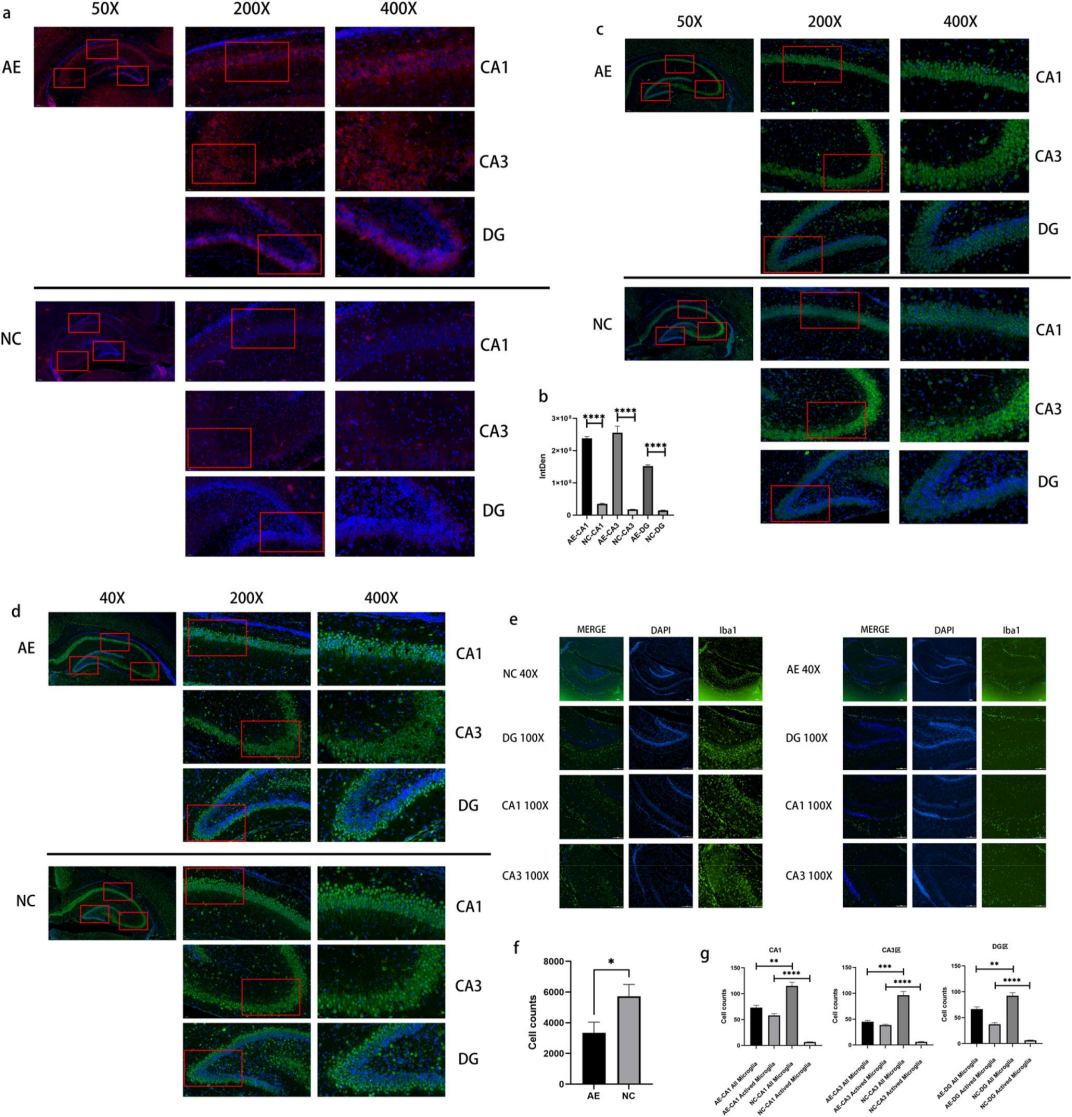
(A) Expression of the CASPASE‐3 protein in the hippocampus after 6 days of alcohol exposure in neonatal rats. CASPASE‐3: red, DAPI: blue. The right images show the areas enclosed by a red box in the left images. (B) Comparison of the fluorescence intensity of CASPASE‐3 after 6 days of alcohol exposure in neonatal rats. The right images show the areas enclosed by a red box in the left images. (C) Expression of NF200 in the rat hippocampus. NF200: green, DAPI: blue. The right images show the areas enclosed by a red box in the left images. (D) Expression of NeuN in the rat hippocampus after alcohol intervention. NeuN: green, DAPI: blue. (E) Expression of Iba1 in the rat hippocampus. Ibal: green, DAPI: blue. (F) Cell expressed NeuN counts in the hippocampus of newborn rats in D. (G) The number of Iba‐1‐positive microglia in the hippocampus of two groups of newborn rats after alcohol intervention was determined. *p < 0.05, **p < 0.01, ***p < 0.001, and ****p < 0.0001.

After neonatal rats were subjected to alcohol exposure for 6 days, we used immunofluorescence to measure the protein expression of CASPASE‐3 in the hippocampus. Figure 7A demonstrates a significant increase in CASPASE‐3 protein expression in the hippocampus of the AE group in comparison to the NC group, particularly in the CA1 and CA3 regions. Further statistical analysis of CASPASE‐3 fluorescence intensity revealed that fluorescence intensity in the DG, CA1, and CA3 regions of the hippocampus was significantly greater in the AE group than in the NC group (*p* < 0.0001) (Figure 7B). These findings suggested that alcohol‐induced cell apoptosis occurred in the DG, CA1, and CA3 regions of the hippocampus in rats.

Immunofluorescence was used to detect the expression of NF200 in the hippocampus of neonatal rats after 6 days of alcohol exposure. The results (Figure 7C) showed that in the NC group, NF200 was interwoven into a net‐like structure within the pyramidal neurons of the hippocampal CA1 and CA3 regions and was anomalously distributed as fine filaments along neuronal axons. The length and location of NF200‐positive fibers in hippocampal CA3 and CA1 neurons were significantly decreased in the AE group compared to the NC group.

We detected the expression of NeuN in the hippocampal tissues of rats to confirm neuronal changes. After continuous alcohol exposure for 6 days in neonatal rats, immunofluorescence staining for NeuN protein in the hippocampus showed loss of hippocampal cells in the AE group, mainly significant loss of neurons in the CA1 and CA3 regions, with a small amount of neuronal loss also observed in the DG region. In the NC group, NeuN immunofluorescence showed a dense and regular arrangement of neurons in all hippocampal regions. Further counting of NeuN‐positive neurons in hippocampal regions revealed that the number of neurons in the AE group (3350 ± 695.6 cells) was markedly lower than that in the NC group (5730 ± 767.8 cells) (*p* < 0.05) (Figure 7D,F).

Brain tissue specimens were collected on PD10 and immunolabeled with Iba1 to observe changes in hippocampal microglia. In the NC group, microglia were distributed in the DG, CA1, and CA3 regions of the hippocampus and exhibited smaller cell bodies and more branches (Figure 7E). The AE group showed a significant decrease in the number of microglia in the DG, CA1, and CA3 regions of the hippocampus compared to the NC group. Additionally, the microglia in the AE group had larger cell bodies, fewer branches, and some spherical forms. Statistical analysis of the immunofluorescence images revealed significant differences in hippocampal microglial distribution between the two groups (*p* < 0.01) (Figure 7G).

### 3.7 Morris water maze

**FIGURE 8:**
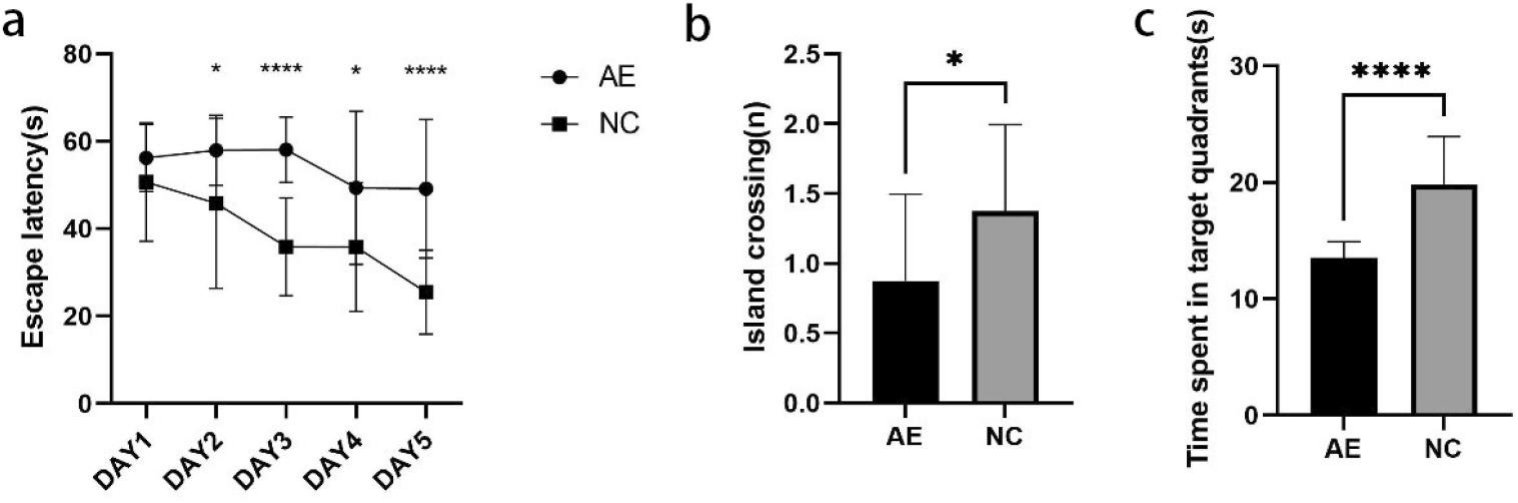
The Morris water maze spatial navigation test. (A) Line graph showing the escape latency of PD28 rats in the acquisition trial for each training day. (B) Bar graphs showing the number of entries into the platform zone, and (C) the time spent in the target quadrants during the probe test. *n* = 16 per group. *p < 0.05, ****p < 0.0001.

Figure 8A demonstrates that in the MWM spatial navigation test, the escape latency of rats in the NC group consistently decreased as the training period rose. Although there was a decreasing trend in escape latency in the AE group, the differences between days were not statistically significant. On training days 2, 3, 4, and 5, the escape latency was significantly longer in adolescent rats in the AE group than in those in the NC group (*p* < 0.05 or *p* < 0.0001). These findings indicate impaired spatial learning abilities in adolescent rats after early postnatal alcohol exposure.

Figure 8B,C show the results of the spatial probe test. Compared to those in the NC group, the numbers of platform crossings in the AE group were markedly lower (*p* < 0.05) and the time spent in the target quadrant was significantly shorter (*p* < 0.0001), indicating impaired spatial memory in rats after early postnatal alcohol exposure.

## 4 DISCUSSION

According to statistics from the World Health Organization, alcohol exposure is one of the major risk factors leading to various health issues, including brain injury. Prenatal alcohol exposure can cause a range of lifelong physiological, mental, behavioral and/or learning disabilities in the fetuses, collectively termed as FASD.^1^, ^2^ The effects of alcohol on the human nervous system are widely believed to vary across developmental stages. Alcohol‐induced injuries to the nervous system can be categorized into three stages based on their effects on structure and function during different developmental periods. The first stage, which lasts for 3 weeks, volves alcohol exposure leading to defects in the neural tube and death of neuroepithelial cells. The second stage occurs between gestational weeks 3 and 20, and is characterised by alcohol exposure primarily causing apoptosis of neuroepithelial cells, abnormal migration of neurons, malformations in the brain and CNS, and anomalies in the corpus callosum. The third stage mainly occurs after 20 weeks of gestation, and is associated with alcohol exposure leading to abnormal brain structures, dysplasia in the cerebellum, microcephaly, altered connections between neurons, and neuronal apoptosis.^3^, ^4^, ^7^, ^8^ Due to the occurrence of significant structural abnormalities as well as microstructural and functional damage, the third stage of alcohol exposure has become the primary area of study for alcohol‐induced prenatal injury to the nervous system. Therefore, we chose PD4–PD9 neonatal rats as an animal model to simulate early postnatal alcohol exposure. Intragastric administration of an 11.9% v/v alcohol–milk solution was implemented in neonatal rats to mimic the impact of alcohol on the third trimester of human brain development.^29^, ^30^ However, we did not include a control group that received only the vehicle without intragastric administration in our study. It is possible that the observed brain developmental abnormalities could be partly attributed to the stress of intragastric administration. Alternatively, the solo intragastric injection may potentially induce malnutrition in neonatal mice, thus leading to brain developmental problems. In future relevant studies, we may consider including a control group that received vehicle without gavage to further clarify the results.

The embryonic stage and early postnatal stage are the critical periods for brain development. During these periods, brain developmental processes are rapid (e.g., synaptogenesis and myelination) and crucial, and the brain has greater developmental plasticity. If adverse external factors impact the brain during these stages, they can lead to impaired brain development and structural and functional abnormalities. Our study revealed that early postnatal alcohol exposure leads to morphological changes in the hippocampus of neonatal rats. After continuous alcohol exposure for 6 days in early postnatal life, reductions in the number of cell layers and cell density were observed in the CA1, CA3, and DG regions of the hippocampus. Various degrees of cell necrosis and vacuolization were also present. Additionally, cytoplasmic eosinophilia, nuclear pyknosis and hyperchromasia were noted in some cells of the CA1 and CA3 regions after alcohol exposure. Furthermore, the immunofluorescence results indicated a significant increase in the expression level of CASPASE‐3 within the hippocampus of neonatal rats in the AE group. Western blot assays revealed increased expression of the apoptosis‐related factors BAX and CASPASE‐3 to varying extents, accompanied by a decrease in the expression level of BCL‐2 in the hippocampus. All the above findings collectively indicate the substantial occurrence of apoptosis in the hippocampal cells of neonatal rats following early postnatal alcohol exposure.

Furthermore, we also found a significant decrease in the number of neurons in the CA1 and CA3 regions of the hippocampus after early postnatal alcohol exposure in rats. NF200 immunostaining revealed shortened, disorganized neuronal projections. These findings indicate that early‐life alcohol exposure leads to selective loss of hippocampal neurons, mainly in the CA1 and CA3 sectors, while surviving neurons exhibit axonal damage.

Although significant differences in the hippocampal structure and apoptosis‐related factor expression after alcohol exposure were observed, all these changes were found 24 h after alcohol consumption ended and may also be induced by withdrawal or hangover effects. Therefore, we performed the MWM test at PD28 (19 days after alcohol exposure was stopped), which showed that the spatial memory of these rats was impaired during early postnatal alcohol exposure. Moreover, compared to normal rats, rats subjected to early postnatal alcohol exposure had a smaller skull size and lower body and brain weights. Hence, we hypothesized that this could be attributed to long‐term damage rather than transient alterations caused by withdrawal or hangover consequences. Nevertheless, additional research is required to confirm the potential long‐term durability of alterations observed in the structure of hippocampal cells or protein levels.

This neuronal and axonal impairment likely underlies the learning and memory deficits observed in juvenile rats after neonatal alcohol exposure. In addition, the water maze test indicated that even brief alcohol exposure during the rapid and critical developmental period could lead to detrimental effects on brain function that persist into adolescence and lead to impaired spatial learning and memory abilities. These findings are consistent with the findings of Wozniak et al., who showed that alcohol exposure during various developmental stages (prenatal, early postnatal, and adolescence) can result in cognitive deficits in experimental animals.^4^

During development, microglia in the hippocampus not only participate in shaping brain structure and neural circuits but also maintains homeostasis of the nervous system throughout life.^31^, ^32^ Immunofluorescence staining revealed that early postnatal alcohol consumption led to microglial activation in the hippocampus, resulting in a decreased quantity compared to the control group. This difference may be associated with the initiation of apoptosis. Hence, we examined the expression of the microglia‐related inflammatory cytokine IL‐6 and apoptotic markers, which were markedly elevated in patients compared to normal controls. Based on these findings, we speculated that the JAK/STAT signaling pathway is involved in neonatal alcohol exposure. To further validate this, we conducted in vitro cell experiments to simulate in vivo alcohol exposure and examined changes in the levels of relevant factors.

In vitro, alcohol intervention in the BV‐2 microglial cell line led to increased secretion of microglia‐derived IL‐6 in the culture medium. These findings indicate that alcohol can induce microglial activation and promote IL‐6 release, consistent with our in vivo findings. Hence, we further treated neurons with IL‐6 and examined JAK signaling activation. We found that IL‐6 induced SOCS3 expression to increase, JAK/STAT activation, pro‐apoptotic BAX and CASPASE‐3 expression to increase to varying degrees, and anti‐apoptotic BCL‐2 expression to decrease, leading to neuronal apoptosis. Subsequently, the application of the JAK inhibitor AG490 was found to inhibit IL‐6‐induced JAK/STAT3 activation and attenuate neuronal apoptotic factor expression.^20^, ^21^, ^22^, ^23^ These findings suggested that AG490 may suppress the IL‐6‐activated JAK/STAT3 pathway and thereby alleviate IL‐6‐induced neuronal apoptosis to some extent. Therefore, this inhibitor could inhibit IL‐6‐induced JAK/STAT signaling and the resulting brain injury caused by alcohol exposure in vivo. AG490 may potentially inhibit brain injury resulting from IL‐6‐induced JAK signaling following alcohol exposure in vivo. Although current research on AG490 has focused primarily on tumor‐related diseases, our findings suggest its potential for improving neurodevelopmental abnormalities caused by early postnatal alcohol exposure. Furthermore, due to its capacity to enhance the advancement of diseases in affected children and maybe reduce the strain on the healthcare system when utilized for therapy, this could broaden our comprehension of the future therapeutic uses of AG490. However, further research is warranted to elucidate the underlying mechanisms of action involved. Furthermore, relevant animal experiments need to be conducted to clarify the specific therapeutic effects of these agents.

Although some interesting results were found, our study has several limitations. First, we investigated the influence of early postnatal alcohol exposure on the development of neonatal rat brains (immature brain). Therefore, we developed alcohol exposure paradigms using neonatal rats in the research, which did not truly simulate the condition of fetal alcohol exposure. In the future, we will attempt to develop alcohol exposure paradigms using pregnant rats to observe the influence of maternal alcohol intake on babies' brain development. Second, as mentioned above, we observed changes in hippocampal structures and apoptosis‐related factors expression levels only at PD10, and further research will be performed to verify whether these changes in structure or protein expression would persist. Third, the protective effects of AG490 were verified only in vitro. A further study will be performed to determine whether AG490 is helpful for alleviating brain damage induced by early postnatal alcohol exposure in vivo.

## 5 CONCLUSION

Our research indicated that early postnatal alcohol exposure could promote microglial cell activation, increase IL‐6 secretion, and subsequently activate the JAK/STAT signaling pathway, leading to hippocampal cell apoptosis. This cascade of events results in delayed growth and development as well as learning and memory impairments in rats. The use of AG490 to inhibit the activation of the JAK/STAT signaling pathway could alleviate the JAK/STAT activation caused by microglial cell activation and subsequent IL‐6 secretion, thus reducing neuronal apoptosis. However, further in vitro studies are needed to investigate the impact of AG490‐mediated inhibition of JAK/STAT signaling pathway activation on structural and functional improvements in rats.

## AUTHOR CONTRIBUTIONS

**Chen Yang**: Conceptualization, methodology, software, validation, formal analysis, investigation, resources, data curation, writing ‐ original draft, writing ‐ review & editing, visualization. **Jianxiong Gui**: Investigation, resources, software, data curation. **Dishue Huang**: Investigation, resources, software. **Ran Ding**: Investigation, resources. **Jie Liu**: Investigation, resources. **Wenjie Zhao**: Investigation, resources. **Jing Yang**: Investigation, resources. **Ziyao Han**: Investigation, resources. **Lingling Xi**e: Investigation, resources. **Xiaoyue Yang**: Investigation, resources, software. **Yanan Pan**: Investigation, resources. **Mingdan Xie**: Investigation, resources. **Li Cheng**: Supervision. **Xiaojie Song**: Funding acquisition, project administration, supervision, writing ‐ review and editing, methodology, Validation. **Li Jiang**: Funding acquisition, project administration, Validation.

## CONFLICT OF INTEREST STATEMENT

The authors declare no conflicts of interest.

## ETHICS STATEMENT

The study was approved by the Ethics Committee of Children's Hospital of Chongqing Medical University.

## Data Availability

Data sharing is not applicable to this article as no new data were created or analyzed in this study.
